# Complete Genome Sequence of *Weissella hellenica* 0916-4-2 and Its Comparative Genomic Analysis

**DOI:** 10.3389/fmicb.2019.01619

**Published:** 2019-07-24

**Authors:** Suresh Panthee, Atmika Paudel, Jochen Blom, Hiroshi Hamamoto, Kazuhisa Sekimizu

**Affiliations:** ^1^Institute of Medical Mycology, Teikyo University, Hachioji, Japan; ^2^Bioinformatics and Systems Biology, Justus-Liebig-University Giessen, Giessen, Germany; ^3^Genome Pharmaceuticals Institute, Bunkyōku, Japan

**Keywords:** *Weissella hellenica*, comparative genomics, secondary metabolism, lactic acid bacteria, probiotic

## Abstract

*Weissella* genus from Leuconostocaceae family forms a group of Gram-positive lactic acid bacteria (LAB) that mostly reside in fermented foods and some have been isolated from the environment and vertebrates including humans. Currently there are 23 recognized species, 16 complete and 37 draft genome assemblies for this genus. *Weissella hellenica* has been found in various sources and is characterized by their probiotic and bacteriocinogenic properties. Despite its widespread importance, little attention has been paid to genomic characterization of this species with the availability of draft assembly of two species in the public database so far. In this manuscript, we identified *W. hellenica* 0916-4-2 from fermented kimchi and completed its genome sequence. Comparative genomic analysis identified 88 core genes that had interspecies mean amino acid identity of more than 65%. Whole genome phylogenetic analysis showed that three *W. hellenica* strains clustered together and the strain 0916-4-2 was close to strain WiKim14. *In silico* analysis for the secondary metabolites biosynthetic gene cluster showed that *Weissella* are far less producers of secondary metabolites compared to other members of Leuconostocaceae. The availability of the complete genome of *W. hellenica* 0916-4-2 will facilitate further comparative genomic analysis of *Weissella* species, including studies of its biotechnological potential and improving the nutritional value of various food products.

## Introduction

The non-spore forming lactic acid bacteria (LAB) of *Weissella* genus within the Leuconostocaceae family contains 23 validly described species. In nature, *Weissella* spp. have been found in a wide range of habitats (Fusco et al., 2015) such as traditional fermented foods, milk, vegetables, feces, environment and vertebrates including humans. In traditional Korean fermented vegetable food kimchi, *Weissella* form the dominant genus at the late stage of fermentation, partly due to their high acid-tolerant property (Kim et al., 2016). Recently, a higher level of interest is being paid for the probiotic, biotechnological and bacteriocinogenic potential of *Weissella*, although some strains are known to act as opportunistic pathogen (Kamboj et al., 2015). Given that most strains regarded as opportunistic pathogens were isolated from the hosts with underlying risk factor, such as immunocompromised condition (Fusco et al., 2015; Kamboj et al., 2015), distinguishing the probiotic and pathogenic *Weissella* has always been a challenging task. Interestingly, the contemporary research has also identified *Weissella* as a producer of botulinum-like toxin (Mansfield et al., 2015; Zornetta et al., 2016). Despite the widespread importance, little attention has been paid to genomic characterization of this genus. This is further evident from the number of genome assemblies available in public database. So far, only 16 species of this genus have been sequenced and complete genome sequence is available for seven species only. kimchi, *Weissella* form the dominant genus at the late stage of fermentation, partly due to their high acid-tolerant property (Kim et al., 2016). Recently, a higher level of interest is being paid for the probiotic, biotechnological and bacteriocinogenic potential of *Weissella*, although some strains are known to act as opportunistic pathogen (Kamboj et al., 2015). Given that most strains regarded as opportunistic pathogens were isolated from the hosts with underlying risk factor, such as immunocompromised condition (Fusco et al., 2015; Kamboj et al., 2015), distinguishing the probiotic and pathogenic *Weissella* has always been a challenging task. Interestingly, the contemporary research has also identified *Weissella* as a producer of botulinum-like toxin (Mansfield et al., 2015; Zornetta et al., 2016). Despite the widespread importance, little attention has been paid to genomic characterization of this genus. This is further evident from the number of genome assemblies available in public database. So far, only 16 species of this genus have been sequenced and complete genome sequence is available for seven species only.

*Weissella hellenica* 0916-4-2 (highlighted in the figures and tables by bold font) was isolated in our laboratory from Korean fermented pickle kimchi and the strain was identified by 16s rRNA sequencing. Although this species harbors a prominent bacteriocinogenic potential, with the production of various bacteriocins such as 7293A (Woraprayote et al., 2015), Weissellicin L (Leong et al., 2013), Weissellicin D (Chen et al., 2014), Weissellicin M (Masuda et al., 2012), and Weissellicin Y (Masuda et al., 2012), there is a great lack of genomic studies among *W. hellenica*. In this manuscript, we completed the genome sequence of *W. hellenica* 0916-4-2 and performed its comparative genomic analysis with publicly available *Weissella* genomes. We found that *W. hellenica* 0916-4-2 clustered with *W. hellenica* Wikim14 based on whole genome phylogeny and harbored two putative genes clusters for the biosynthesis of bacteriocin and non-ribosomal peptide synthetase.

## Materials and Methods

### Strain and DNA Extraction

The strain used in this study was *W. hellenica* 0916-4-2 isolated from Korean pickle kimchi using MRS medium. Genomic DNA was isolated as explained (Panthee et al., 2017b).

### Whole Genome Sequencing

The library preparation for Oxford Nanopore MinION and Thermo Fisher Ion PGM sequencing was performed as explained previously (Panthee et al., 2017a, c, 2018). Briefly, for ultra-long reads library, 1 μg genomic DNA was end-prepped using the NEBNext End repair/dA-tailing Module (New England Biolabs, Inc., Ipswich, MA, United States) and the DNA was cleaned up using Agencourt AMPure XP (Beckman Coulter Inc., Brea, CA, United States). The DNA was then ligated to the adapter using NEB Blunt/TA Ligase Master Mix (NEB). The library was purified using Agencourt AMPure XP (Beckman Coulter Inc) and reads were obtained using the 48-h protocol and live base-calling after loading the library to a primed FLO-MIN106 R9.4 SpotON Flow Cell. The 400 base-reads was prepared after fragmentation of 100 ng of the DNA using the Ion Xpress^TM^ Plus Fragment Library Kit (Thermo Fisher Scientific, Waltham, MA, United States). The libraries were enriched in an Ion ^TM^318 Chip v2 using Ion Chef (Thermo Fisher Scientific), and subsequent sequencing was performed in the Ion PGM System (Thermo Fisher Scientific).

### Read Correction and Genome Assembly

Read correction and genome assembly was performed as explained previously (Panthee et al., 2018). We obtained 2M reads (mean length 277 bp) from Ion PGM, and 247K reads from MinION (mean length 7 kb), accounting for approximately 273-fold and 900-fold genome coverage, respectively. The high quality MinION long reads were filtered using Filtlong and self-correction and trimming was performed using canu 1.7 (Koren et al., 2017). The short reads from Ion PGM were corrected using SPADES 3.11 (Bankevich et al., 2012). The hybrid error correction of long reads was then performed using LoRDEC (Salmela and Rivals, 2014). The final genome assembly was performed from the long corrected reads using Flye 2.3.3 assembler (Kolmogorov et al., 2018). Further polishing of the assembly was performed by mapping the short reads to the assembly followed by consensus generation. The assembled genome was annotated using the NCBI Prokaryotic Genome Annotation Pipeline (PGAP) to find 1778 protein coding genes and 101 RNA genes. The genome was further submitted to PathogenFinder (Cosentino et al., 2013) to predict the absence of pathogenic genes. Functional analysis of the protein coding genes was performed in Blast2GO ver 5.1.13 (BioBam Bioinformatics, Valencia, Spain).

### Genomic Data of Other *Weissella* Species and Comparative Genomic Analysis

Genomic data of the additional *Weissella* species analyzed in this study were obtained from the NCBI. The accession numbers and assembly status are indicated in the Supplementary Table S1. To construct the phylogenetic tree, first the core genes of these genomes were computed, and the alignment of each core gene set was generated using MUSCLE, and the alignments were concatenated to create a single alignment. This alignment was used to generate the phylogenetic tree using neighbor-joining algorithm in EDGAR (Blom et al., 2016). The core- and pan-proteomes were predicted using EDGAR (Blom et al., 2016). The COG analysis of the core proteome was performed using eggNOG-Mapper (Huerta-Cepas et al., 2017). The analysis of secondary metabolite gene clusters was performed as explained (Panthee et al., 2017b) using antiSMASH (Blin et al., 2017).

### *Weissella* Virulence Factors Analysis

For the bioinformatic analysis of virulence factors in *Weissella* genomes, we downloaded the amino acid sequence of core virulence factors from the virulence factor database (Chen et al., 2005; Liu et al., 2018) and performed a BLAST search against the *Weissella* proteome with a cut off e-value of e^–30^ and minimum identity of >70%. To analyze the virulence potential of *W. hellenica* 0916-4-2 in a mouse model, bacteria was cultivated overnight in MRS at 30°C. The culture was centrifuged and resuspended in phosphate-buffered saline (PBS). Six weeks old female ICR mice (*n* = 5) were injected with bacteria equivalent to 200 μl of the overnight culture (1.5 × 10^9^ CFU) through intraperitoneal route and survival was observed for 5 days. All mouse experimental protocols were approved by the Animal Use Committee at the Genome Pharmaceuticals Institute.

## Results and Discussion

### Assembly and Analysis of the *W. hellenica* 0916-4-2 Genome

**FIGURE 1:**
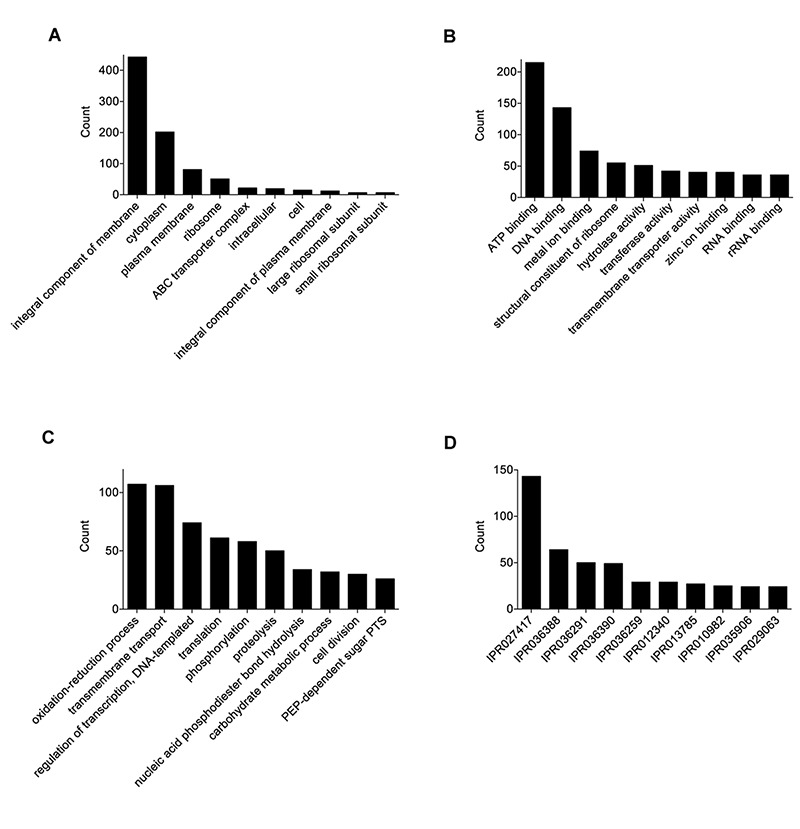
Functional annotation and analysis of *W. hellenica* 0916-4-2 genome. Gene Ontology (GO) term counts for: **(A)** cellular component, **(B)** molecular functions, and **(C)** biological processes. **(D)** InterPro scan for gene families.

The final 0916-4-2 assembly had a genome size of 1.93 Mb with a circular chromosome and two plasmids (Table 1). A total of 4828 Gene Ontology (GO) terms were assigned to 1503 (85%) genes, which included 1703 (35%), 2193 (46%), and 932 (19%) GO annotations for the biological process, molecular function, and cellular component category, respectively (Figures 1A–C). The most abundant GO annotation included oxidation-reduction process, ATP binding, and integral component of the membrane, respectively. InterProScan showed that a total of 3132 families were assigned for 1585 (89%) of the total proteins, where P-loop containing nucleoside triphosphate hydrolase family (IPR027417) was the highest 143 (4.6%) (Figure 1D). Given that some of the *Weissella* species harbored the genes for drug resistance (Abriouel et al., 2015), none were detected in the genome of the 0916-4-2 strain.

**TABLE 1:** General feature(s) of *Weissella hellenica* 0916-4-2 genome.

**Feature**	**Characteristics**
Genome size	1,937,540 bp
Number of chromosomes	1
Number of plasmids	2
GC content (%)	36.9
Chromosome length	1,875,603 bp
Plasmid 1 pWHSP041 length	41,289 bp
Plasmid 2 pWHSP020 length	20,648 bp
Protein coding genes	1778
RNA genes	101

### General Features and Comparative Genomics of *Weissella* Species

**FIGURE 2:**
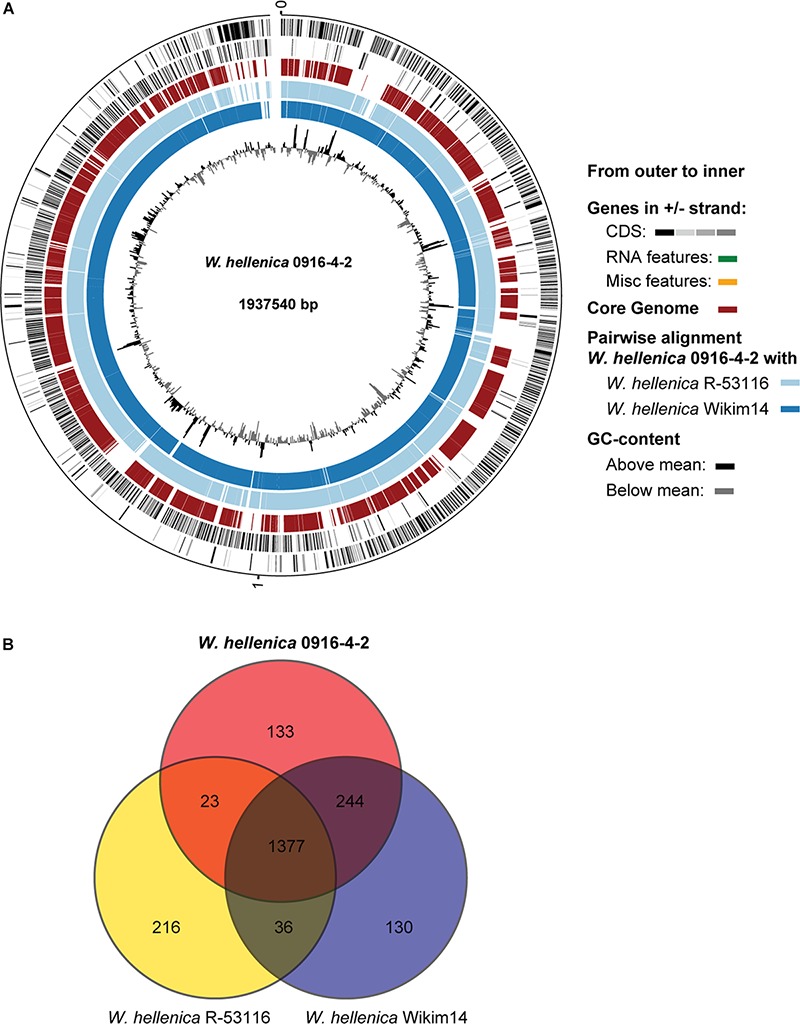
Comparative genomic analysis of *W. hellenica*. **(A)** Circular genome comparison showing the core genome, pairwise alignment, and GC content. The meaning of each circle is indicated by the legend in the figure. **(B)** Venn diagram showing shared and unique genes among *W. hellenica*s.

**FIGURE 3:**
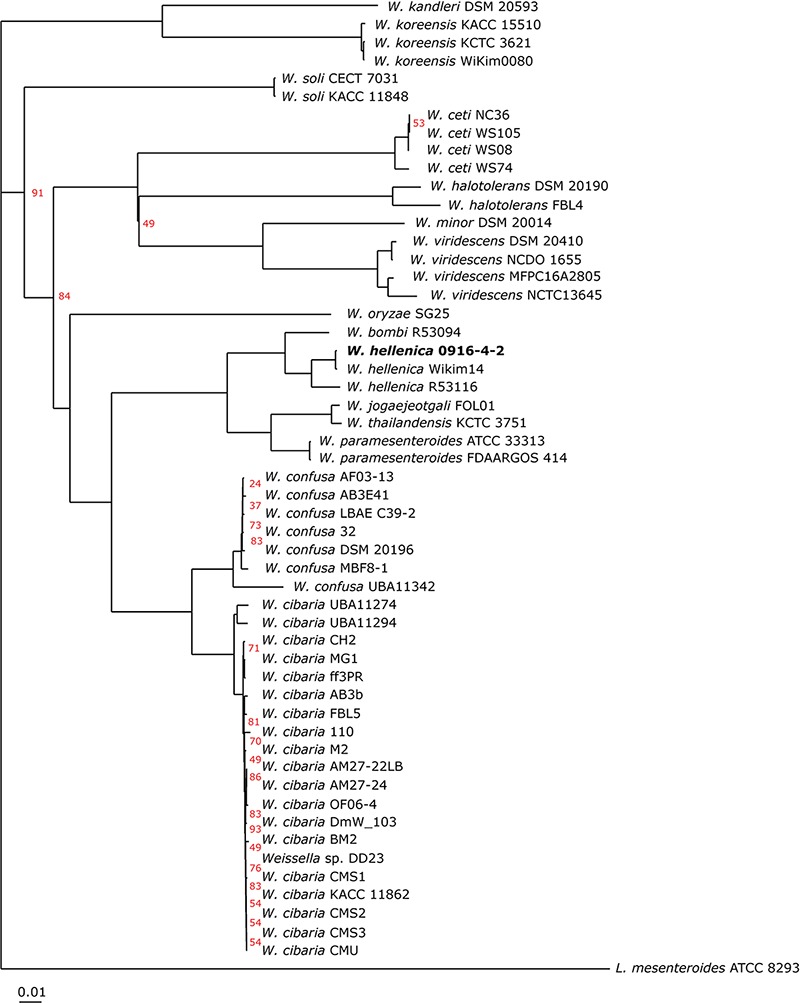
Phylogeny of *Weissella* species based on single-copy core orthologs. *W. hellenica* 0916-4-2 proteome, along with 52 *Weissella* and *Leuconostoc mesenteroides* ATCC 8293 proteomes obtained from public database and core genome was computed. The alignment of each core gene set was generated using MUSCLE, and the alignments were concatenated to create a single alignment. This alignment was used to generate the phylogenetic tree using neighbor-joining algorithm in EDGAR using *L. mesenteroides* ATCC 8293 as an outgroup. Bootstrap conservation values are shown in percent out of 200 iterations. Branches without support value showed 100% bootstrap support. Tree for 54 genomes, built out of a core of 67 genes with 20037 AA-residues per genome, 1081998 in total.

As a first step toward the comparative analysis of the *W. hellenica* 0916-4-2 genome, we analyzed the gene sets with *W. hellenica* R-53116 and Wikim14 genomes using pairwise alignment (Figure 2A) and identified the strain-specific and commonly shared genes (Figure 2B). Based on the analysis, we found that 1377 genes were distributed throughout the strains; the 0916-4-2 genome had a larger set of genes shared with Wikim14 genome compared to R-53116 genome and the number of genes unique to 0916-4-2 was 133. Interestingly, nearly half of the unique genes were regarded either hypothetical protein or domain of unknown function harboring proteins. Next, to provide an overview of *Weissella* genus and perform a comparative genomic analysis, the sequence data of 53 *Weissella* strains, that included 16 complete genome sequence assemblies, was obtained from NCBI. Of the 23 recognized species as of December 2018, these genomes represent only 16 species and complete genome is available only for seven species: *W. ceti*, *W. cibaria*, *W. jogaejeotagli*, *W. koreensis*, *W. paramesenteroides*, and *W. soli.* The assembly accession numbers and status of assemblies are shown in Supplementary Table S2. To determine the genomic variability between *Weissella* species, we performed the comparative genomic analysis of all the genome assemblies. The set of commonly shared genes, core genes, in the genus was determined using EDGAR (Blom et al., 2016) and the whole genome phylogenetic tree was constructed. The phylogenetic tree indicated that this analysis was capable of grouping the various strains of a single species into a single cluster (Figure 3). *W. hellenica* 0916-4-2 clustered with *W. hellenica* Wikim14, suggesting that these two strains might harbor similar biological properties. Mean AAI analysis of the core proteome showed that interspecies similarity was higher than ∼65% among all the species whereas the value was much higher for any of the *W. bombi*, *W. hellenica*, *W. paramesenteroides*, *W. jogaejeotgali*, *W. thailandensis*, *W. cibaria*, and *W. confusa* pair. Intraspecies similarity was further higher with more than 97% for all the species (Table 2 and Supplementary Table S2). We found that all the strains of *W. ceti*, *W. soli* and *W. paramesenteroids* and some strains of *W. cibaria* harbored 100% intraspecies similarity (Supplementary Table S1). Among the interspecies similarity, a high degree of similarity was obtained for *W. jogaejeotgali* FOL01 and *W. thailandensis* KCTC3751 with a 99% mean AAI (Table 2).

**TABLE 2:** Heatmap of the percentage AAI similarity between the conserved regions of the genus *Weissella*.

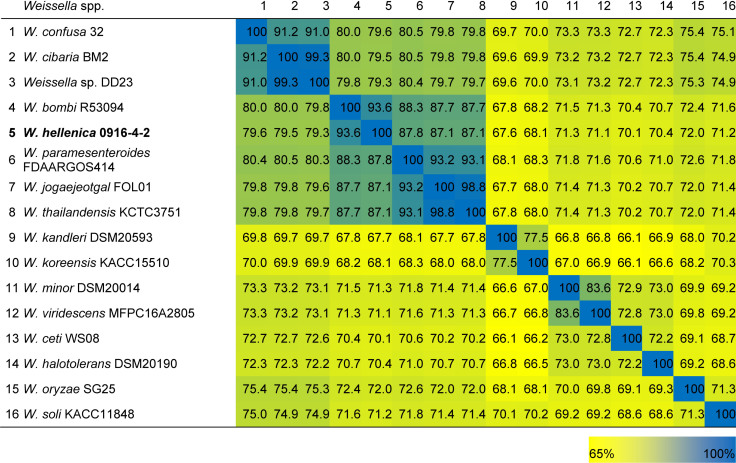

*Species on the first column and first row of the table are represented in the same numeric order. The numbers show the percentage similarity between the conserved regions of the genomes, where the colors vary from yellow (low similarity) to blue (high similarity).*

The members of *Weissella* genus are heteroformentative (Collins et al., 1993) attributed to the lack of phosphofructokinase (Sun et al., 2015). We did not find homologs for phosphofructokinase and lactate dehydrogenase in the 0916-4-2 genome. This suggested that 0916-4-2 lacks ability to produce L-lactate and this was further consistent with the observation of multiple copies of D-2-hydroxyacid dehydrogenase gene responsible for metabolism of pyruvate through D-lactate pathway. An analysis of *Weissella* genomes indicated that majority of *Weissella* had the genes for D-lactate formation with *W. viridescens*, *W. minor*, *W. confusa*, *W. cibaria*, and *Weissella* sp. DD23 harboring the genes for the production of both D/L configuration of lactic acid (Table 3). This might provide a possible explanation for the presence of significant amount of D-lactic acid (Yoon et al., 2005) and increase in lactic acid content at late stage of kimchi fermentation (You et al., 2017).

**TABLE 3:** Genes involved in D/L-lactate fermentation in *Weissella* genomes.

**S. N.**	***Weissella***	**Genes fermenting (no of genes)**	
		**L-lactate**	**D-lactate**
1	*W. bombi* R-53094	−	+ (5)
2	*W. ceti* NC36	−	+ (1)
3	*W. ceti* WS08	−	+ (1)
4	*W. ceti* WS105	−	+ (1)
5	*W. ceti* WS74	−	+ (1)
6	*W. cibaria* AB3b	+ (2)	+ (2)
7	*W. cibaria* AM27-22LB	+(2)	+ (2)
8	*W. cibaria* AM27-24	+ (2)	+ (2)
9	*W. cibaria* BM2	+ (2)	+ (2)
10	*W. cibaria* CH2	+ (2)	+ (2)
11	*W. cibaria* CMS1	+ (2)	+ (2)
12	*W. cibaria* CMS2	+ (2)	+ (2)
13	*W. cibaria* CMS3	+ (2)	+ (2)
14	*W. cibaria* CMU	+ (2)	+ (2)
15	*W. cibaria* DmW103	+ (2)	+ (2)
16	*W. cibaria* ff3PR	+ (2)	+ (2)
17	*W. cibaria* KACC11862	+ (2)	+ (2)
18	*W. cibaria* M2	+ (2)	+ (2)
19	*W. cibaria* MG1	+ (2)	+ (2)
20	*W. cibaria* OF06-4	+ (1)	+ (1)
21	*W. cibaria* strain 110	+ (2)	+ (2)
22	*W. cibaria* strain FBL5	+ (1)	+ (2)
23	*W. cibaria* UBA11274	+ (1)	+ (2)
24	*W. cibaria* UBA11294	+ (1)	+ (1)
25	*W. confusa* 32	+ (1)	+ (2)
26	*W. confusa* AB3E41	+ (1)	+ (2)
27	*W. confusa* AF03-13	+ (1)	+ (2)
28	*W. confusa* DSM20196	+ (1)	+ (2)
29	*W. confusa* LBAE C39-2	+ (1)	+ (1)
30	*W. confusa* MBF8-1	+ (1)	+ (1)
31	*W. confusa* UBA11342	+ (1)	+ (2)
32	*W. halotolerans* DSM20190	+ (1)	+ (3)
33	*W. halotolerans* FBL4	+ (1)	+ (3)
**34**	***W. hellenica* 0916-4-2**	**−**	**+ (5)**
35	*W. hellenica* R-53116	−	+ (5)
36	*W. hellenica* WiKim14	−	+ (5)
37	*W. jogaejeotgali* FOL01	−	+ (6)
38	*W. kandleri* DSM20593	−	+ (3)
39	*W. koreensis* KACC15510	−	+ (3)
40	*W. koreensis* KCTC3621	−	+ (3)
41	*W. koreensis* WiKim0080	−	+ (3)
42	*W. minor* DSM20014	+ (1)	+ (3)
43	*W. oryzae* SG25	−	+ (3)
44	*W. paramesenteroides* ATCC33313	−	+ (7)
45	*W. paramesenteroides* FDAARGOS414	−	+ (7)
46	*W. soli* CECT7031	−	+ (5)
47	*W. soli* KACC11848	−	+ (5)
48	*Weissella* sp. DD23	+ (2)	+ (2)
49	*W. thailandensis* KCTC3751	−	+ (4)
50	*W. viridescens* DSM20410	+ (1)	+ (2)
51	*W. viridescens* MFPC16A2805	+ (1)	+ (2)
52	*W. viridescens* NCDO1655	+ (1)	+ (2)
53	*W. viridescens* NCTC13645	+ (1)	+ (3)

*–, Absent; +, present.*

*W. hellenica* was first described in 1993 where Collins et al. (1993) reported the inability of this species to utilize various carbohydrates including D-cellobiose, D-raffinose, and gentiobiose for acid production. We analyzed the carbohydrate utilization pattern and found that *W. hellenica* 0916-4-2 can utilize D-cellobiose and D-raffinose very well; and gentiobiose partially (Supplementary Figure S1). This indicated the variation in strain specific properties of *W. hellenica* toward carbohydrate fermentation. Moreover, the metabolism of D-cellobiose and D-raffinose was consistent with the presence of the phosphotransferase system with β-glucosidase and α-galactosidase genes in the genome. Raffinose, present in various cruciferous vegetables including cabbage (Santarius and Milde, 1977), is not metabolized by humans and results in various intestinal disorders like flatulence (Rackis, 1981). Our study indicates that vegetable fermentation using 0916-4-2 strain might enhance the nutritional value of the products such as kimchi.

### Core Genome Analysis of *Weissella* Species

**FIGURE 4:**
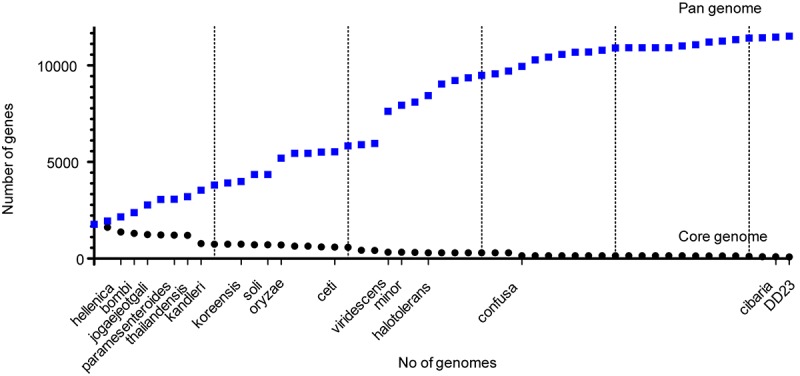
Core and pan proteome analysis of *Weissella*.

Among the 53 *Weissella* strains analyzed, the core and pan genome size was 88 and 11,519, respectively (Figure 4). Among the 88 core genes in *Weissella*, three were found to be categorized as hypothetical and a COG analysis of the remaining 85 genes showed that more than a third of the genes were categorized to be involved in translation and nearly 13% of the genes were categorized to have unknown function (Table 4). Further, with addition of each species, there was a significant change in the number of core and pan genome suggesting a great genomic variation among the species within genus which could be a result of genomic fluidity or significant gene gain/loss during adaptation in natural niche.

**TABLE 4:** COG analysis of core *Weissella* proteome.

**Code**	**Description**	**Number of genes**
C	Energy production and conversion	5
D	Cell cycle control and mitosis	1
E	Amino Acid metabolism and transport	3
F	Nucleotide metabolism and transport	4
G	Carbohydrate metabolism and transport	2
I	Lipid metabolism	3
J	Translation	29
K	Transcription	5
L	Replication and repair	4
M	Cell wall/membrane/envelop biogenesis	2
N, U	Cell motility, Intracellular trafficking and secretion	1
O	Post-translational modification, protein turnover, chaperone functions	5
P	Inorganic ion transport and metabolism	3
Q	Secondary Structure	1
T	Signal Transduction	3
U	Intracellular trafficking and secretion	3
S	Function Unknown	11

### Virulence Factors in *Weissella* Genome and *W. hellenica* 0916-4-2

Based on the BLAST search against the core virulence factors from the virulence factor database (Chen et al., 2005; Liu et al., 2018), we did not find homologs associated with toxin production system, including botulinum neurotoxin homolog from *W. oryzae* SG25 (Mansfield et al., 2015; Zornetta et al., 2016), in the analyzed *Weissella* genomes. We detected some of the genes, *hasC* (UDP-glucose pyrophosphorylase); *cpsI* (UDP-galactopyranose mutase); *gnd* (6-phosphogluconate dehydrogenase); *cpsF* and *clpE* (ATP-dependent protease) involved in virulence in various bacteria. These genes constitute a part of a pathway, indicating that the existence of a single genes is not sufficient to exert virulence. This suggested the need of a detailed investigation into the role of these genes in functional *Weissella* trait. Interestingly, all but *Weissella* sp. DD23 harbored *hasC* and there was no occurrence of species-specific virulence gene. We further expanded our study by the examination of the pathogenicity of *W. hellenica* 0916-4-2 through intraperitoneal injection to five mice. We found that mice did not die for 5 days post-injection suggesting its non-pathogenicity (data not shown). Although some members of the genus *Weissella* are opportunistic pathogen, there is a lack of clear demarcation between the probiotic and pathogenic strains among the strains analyzed in this report suggesting a need of a detailed investigation regarding *Weissella* pathogenicity.

### Bacteriophages and Phage Defense

**FIGURE 5:**
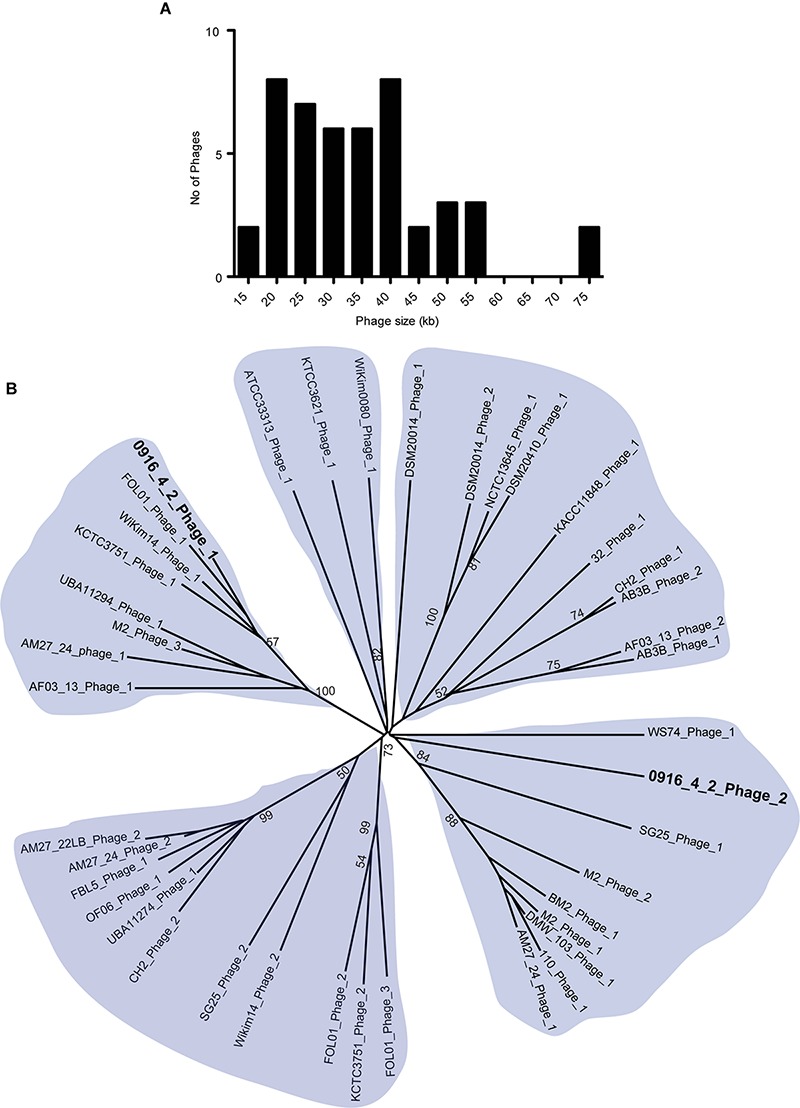
Analysis of *Weissella* phages. Histogram of length **(A)** and radial phylogenetic tree **(B)** of the complete phages identified in *Weissella*. The phages were analyzed using VICTOR (Meier-Kolthoff and Göker, 2017) and pairwise comparisons of the nucleotide sequences were conducted using the Genome-BLAST Distance Phylogeny method under settings recommended for prokaryotic viruses. The numbers above branches are the bootstrap support values from 100 replications (values ≥50 are shown).

Phages are critical part of bacterial genome that facilitate various beneficial traits for bacteria such as adaptation in new environmental niches and acquisition of bacterial resistance. We used PHASTer (Arndt et al., 2016) to search for putative prophage elements in the complete and draft *Weissella* genomes to identify a total of 127 phages, of which 44 were considered as intact (Supplementary Table S1) and the number of phages in each strain ranged from 1 to 9. The large number of incomplete phages might be due to the draft nature of the assembly, as the phages generally have the repeated sequences. The intact phages from *Weissella*, ranged from 14 to 74 kb in length and majority had a length of 20–40 kb (Figure 5A). To identify the presence of potential pathogenic genes in the intact phages, we looked for the possible presence of virulence factors and antibiotic resistance genes. We did not find any virulence factors in the phages and a search in the CARD database (McArthur et al., 2013) using perfect and strict hits did not find any genes responsible for drug resistance. All the intact phages were further analyzed using Victor (Meier-Kolthoff and Göker, 2017) and phylogenetic tree was created. Based on the analysis, we grouped the phages in five groups. Our finding suggested that among the two intact phages present in *W. hellenica* 0916-4-2, one clustered with the phage from *W. jogaejeotgali* FOL01 and the next one fell onto relatively distinct cluster (Figure 5B). Interestingly, we found that two separate strains of *W. soli* (CECT7031 and KACC11848) and *W. cibaria* (AM27-22LB and AM27-24) shared a common phage, suggesting a very close intraspecies relationship.

### Secondary Metabolic Potential

The genome assemblies were analyzed using antiSMASH (Blin et al., 2017) for the possible presence of gene clusters encoding for secondary metabolites. We found a total of 10 predicted secondary metabolites classified as: arylpolyene (1), bacteriocin (4), lassopeptide (2), NRPS (1), and thiopeptide (2) (Table 5). Given that only 9 out of 53 strains harbored the gene clusters for secondary metabolites, the genus *Weissella* can be regarded as a low producer of secondary metabolites. Further, this data was compared with other families of the order Lactobacillales. The genome assemblies were downloaded from NCBI and bioinformatic analysis for the presence of secondary metabolite biosynthetic gene clusters was performed. Bacteriocin constituted the major class of metabolite predicted to be present in the genome. Furthermore, in contrast to *Weissella*, the other members of Leuconostocaceae family were found to harbor about one secondary metabolite gene cluster per genome (Table 6). Among all the Lactobacillales, *Lactococcus* and *Streptococcus* were found to be relatively higher producers of metabolites with the presence of about three gene clusters per genome.

**TABLE 5:** Secondary metabolic potential of *Weissella* species.

**S. N.**	***Weissella***	**Secondary metabolites predicted**
1	*W. cibaria* AB3b	1
2	*W. cibaria* strain 110	1
**3**	***W. hellenica* 0916-4-2**	**2**
4	*W. hellenica* Wikim14	1
5	*W. minor* DSM20014	1
6	*W. paramesenteroides* ATCC33313	1
7	*W. paramesenteroides* FDAARGOS414	1
8	*Weissella* sp. DD23	1
9	*W. viridescens* NCTC13645	1

**TABLE 6:** Secondary metabolic potential of Lactobacillales.

**Family**	**Genus**	****	**Secondary metabolite gene clusters identified**																	
		**Genomes analyzed**	**Total**	**Bacteriocin**	**Terpene**	**-Peptide**				**-Lactone**			**NRPS**	**PKS/NRPS**	**RiPP**	**Ladderane**	**LAP**	**Aryl polyene**	**Furan**	**PKS**
						**Lanthi**	**Lasso**	**Thio**	**Sacti**	**Hser**	**Beta**	**Butyro**								
Aerococcaceae	*Abiotrophia*	2	0																	
	*Aerococcus*	65	22	4	12	1	2			1			2							
	*Dolosicoccus*	3	0																	
	*Eremococcus*	2	0																	
	*Facklamia*	11	6	2	1	2	1													
	*Globicatella*	4	5	1							4									
	*Ignavigranum*	1	0																	
Carnobacteriaceae	*Agitococcus*	1	2	2																
	*Alkalibacterium*	9	8		7					1										
	*Allofustis*	1	0																	
	*Alloiococcus*	2	2	2																
	*Atopobacter*	2	4	1		2							1							
	*Atopococcus*	1	0																	
	*Atopostipes*	1	1		1															
	*Carnobacterium*	47	78	35	19	4	1	1		3			15							
	*Desemzia*	1	2		2															
	*Dolosigranulum*	12	19	14		5														
	*Granulicatella*	11	1			1														
	*Isobaculum*	1	0																	
	*Jeotgalibaca*	5	8		5	2		1												
	*Lacticigenium*	1	0																	
	*Marinilactibacillus*	5	8	3	5															
	*Pisciglobus*	1	2		2															
	*Trichococcus*	20	9	2					7											
Enterococcaceae	*Bavariicoccus*	2	4	2	2															
	*Catellicoccus*	1	0																	
	*Enterococcus*	149	239	202	18	14	1				1				1		1	1		
	*Melissococcus*	18	2			2														
	*Pilibacter*	1	3	2		1														
	*Tetragenococcus*	26	25		25															
	*Vagococcus*	21	12	2	4	2	1					1			1	1				
Lactobacillaceae	*Lactobacillus*	35	22	9	3	5						1	1				2	1		
	*Pediococcus*	26	5	4		1														
	*Sharpea*	10	4						4											
Leuconostocaceae	*Convivina*	1	1		1															
	*Fructobacillus*	9	6	6																
	*Leuconostoc*	26	47	20	5	1					17							1	2	1
	*Oenococcus*	15	15	5	4	1											5			
	***Weissella***	**53**	**10**	**4**			**2**	**2**					**1**					**1**		
Streptococcaceae	*Floricoccus*	2	0																	
	*Lactococcus*	47	167	53		10					39	1	1	4	56		1	1	1	
	*Lactovum*	0																		
	*Okadaella*	0																		
	*Streptococcus*	28	76	56		3							5	1	6		3		1	1

## Summary

The finished genome of *W. hellenica* 0916-4-2 is 1.93 Mb with a chromosome and two plasmids. We found that this strain can utilize D-cellobiose, a cellulose derivative, and harbored two putative secondary metabolite biosynthetic gene clusters. The ability of 0916-4-2 to utilize raffinose can be exploited to enhance the nutritional value of fermented vegetables. Comparative genomic analysis revealed a high degree of genomic variation among *Weissella* species. In recent post-genomic era, although the genome sequence data are becoming increasingly available for diverse bacterial species including *Weissella*, a species which has both probiotic and opportunistic pathogenic potential, the future research should focus on the detailed investigation of the genome and the association to specific gene(s) to the functional traits.

## Data Availability

The complete genome assembly of *Weissella hellenica* 0916-4-2 has been deposited at DDBJ/ENA/GenBank with accession numbers: CP033608, CP033609, and CP033610 for chromosome, pWHSP041, and pWHSP020, respectively. The BioProject accession number for this project is: PRJNA503947.

## Author Contributions

SP, HH, and KS designed the study. SP and AP performed the genome sequencing and annotation. SP, AP, and JB performed the comparative genomic analysis. SP and AP wrote the manuscript. HH and KS integrated the research and critically revised the manuscript for important intellectual content. KS approved the final version of the manuscript.

## Conflict of Interest Statement

The authors declare that the research was conducted in the absence of any commercial or financial relationships that could be construed as a potential conflict of interest.
