# Supplementary material for: Complete Genome Sequence of Weissella hellenica 0916-4-2 and Its Comparative Genomic Analysis
Source: Front Microbiol. 2019 Jul 24;10:1619. doi: 10.3389/fmicb.2019.01619 (PMC6667553; doi:10.3389/fmicb.2019.01619)
Supplement: Supplementary file 1 [file Data_Sheet_1.pdf]

Supplementary information to:

Complete genome sequence of *Weissella hellenica* 0916-4-2 and its comparative genomic analysis

Suresh Panthee[1], Atmika Paudel[1], Jochen Blom[2], Hiroshi Hamamoto[1], Kazuhisa Sekimizu[1,3,*]

[1]Teikyo University Institute of Medical Mycology, 359 Otsuka, Hachioji, Tokyo 192-0395, Japan.

[2]Bioinformatics and Systems Biology, Justus-Liebig-University Giessen, 35392 Giessen, Hesse, Germany

[3]Genome Pharmaceuticals Institute, 102 Next Building, 3-24-17 Hongo, Bunkyo-ku, Tokyo 113-0033, Japan

* Corresponding author: sekimizu@main.teikyo-u.ac.jp

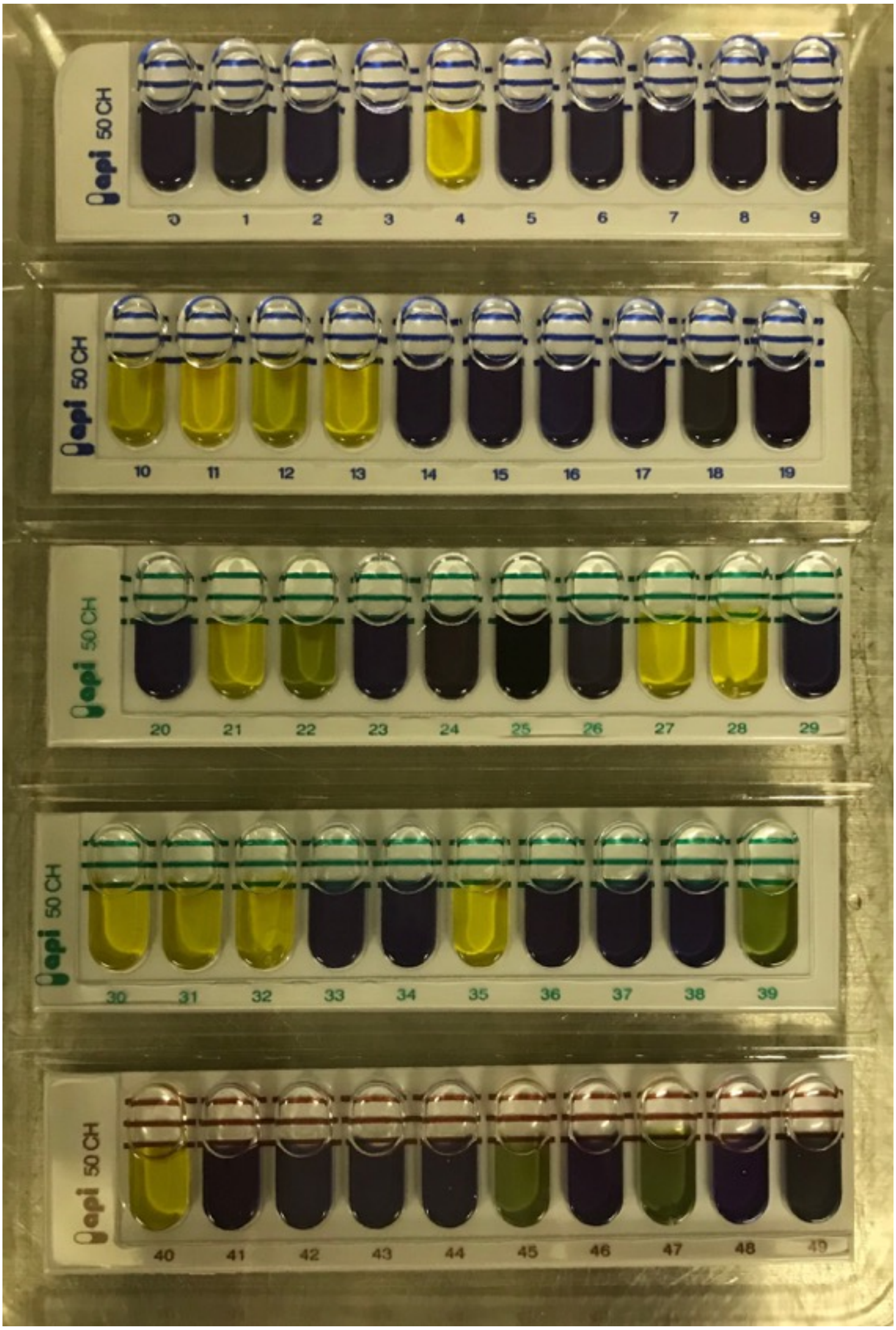

Figure S1. Analysis of carbohydrate metabolism by *W. hellenica* 0916-4-2 using API 50 CH from BioMerieux (Marcy-l'Etoile, France).

Table S1. Genome assembly and number of phages identified in each genome. Phages in FBL4 were not analyzed due to the anomalous nature of the assembly.

SN	Weissella	Assembly		Phage(s)	
		Accession	status	Total	intact
1	*W. bombi* R-53094	GCA_900094835.1	draft	1	
2	*W. ceti* NC36	GCA_000320345.1	draft	2	
3	*W. ceti* WS08	GCA_000732905.1	complete	2	
4	*W. ceti* WS105	GCA_000750535.1	complete	2	
5	*W. ceti* WS74	GCA_000750515.1	complete	1	1
6	*W. cibaria* AB3b	GCA_000878185.1	draft	2	2
7	*W. cibaria* AM27-22LB	GCA_003469985.1	draft	4	3
8	*W. cibaria* AM27-24	GCA_003469955.1	draft	4	3
9	*W. cibaria* BM2	GCA_003010455.1	complete	3	1
10	*W. cibaria* CH2	GCA_001308145.2	complete	3	2
11	*W. cibaria* CMS1	GCA_002243305.1	complete	1	
12	*W. cibaria* CMS2	GCA_001932695.1	complete	1	
13	*W. cibaria* CMS3	GCA_001951095.1	complete	1	
14	*W. cibaria* CMU	GCA_001951075.2	complete	1	
15	*W. cibaria* DmW103	GCA_002115575.1	draft	2	1
16	*W. cibaria* ff3PR	GCA_000878215.1	draft	1	
17	*W. cibaria* KACC11862	GCA_000193635.2	draft	2	
18	*W. cibaria* M2	GCA_003076495.1	complete	4	
19	*W. cibaria* MG1	GCA_000878205.1	draft	1	3
20	*W. cibaria* OF06-4	GCA_003439565.1	draft	1	
21	*W. cibaria* strain 110	GCA_002614625.1	draft	1	1
22	*W. cibaria* strain FBL5	GCA_001614295.1	draft	3	1
23	*W. cibaria* UBA11274	GCA_003535615.1	draft	1	1
24	*W. cibaria* UBA11294	GCA_003542855.1	draft	4	1
25	*W. confusa* 32	GCA_003284505.1	draft	3	1
26	*W. confusa* AB3E41	GCA_900166935.1	draft	4	1
27	*W. confusa* AF03-13	GCA_003466065.1	draft	3	
28	*W. confusa* DSM20196	GCA_001436895.1	draft	2	2
29	*W. confusa* LBAE C39-2	GCA_000239955.2	draft	4	
30	*W. confusa* MBF8-1	GCA_001884305.1	draft	1	
31	*W. confusa* UBA11342	GCA_003512765.1	draft	2	
32	*W. halotolerans* DSM20190	GCA_001436865.1	draft	3	
33	*W. halotolerans* FBL4	GCA_001614305.1	Draft/anomalous	-	-
34	*W. hellenica* 0916-4-2	This paper	complete	3	2
35	*W. hellenica* R-53116	GCA_900095015.1	draft	2	
36	*W. hellenica* WiKim14	GCA_000715455.1	draft	3	2
37	*W. jogaejeotgali* FOL01	GCA_001932615.1	complete	6	3
38	*W. kandleri* DSM20593	GCA_001438705.1	draft	2	
39	*W. koreensis* KACC15510	GCA_000219805.1	complete	1	
40	*W. koreensis* KCTC3621	GCA_000277645.1	draft	4	1
41	*W. koreensis* WiKim0080	GCA_002950695.1	complete	2	1
42	*W. minor* DSM20014	GCA_001437425.1	draft	3	2
43	*W. oryzae* SG25	GCA_000691805.2	draft	5	2
44	*W. paramesenteroides* ATCC33313	GCA_000160575.1	draft	2	1
45	*W. paramesenteroides* FDAARGOS414	GCA_002386265.1	complete	1	
46	*W. soli* CECT7031	GCA_003353445.1	draft	2	1
47	*W. soli* KACC11848	GCA_001761545.1	complete	2	1
48	*Weissella* sp. DD23	GCA_001579105.1	draft	3	
49	*W. thailandensis* KCTC3751	GCA_003363255.1	draft	3	2
50	*W. viridescens* DSM20410	GCA_001437355.1	draft	1	1
51	*W. viridescens* MFPC16A2805	GCA_900216215.1	chromosome	1	
52	*W. viridescens* NCDO1655	GCA_001460875.1	draft	2	
53	*W. viridescens* NCTC13645	GCA_900460455.1	draft	9	1

Table S2 (related to Table 2) Heatmap of the percentage AAI similarity between the conserved regions of the genus *Weissella*. Species on the first column and first row of the table are represented in the same numeric order. The numbers show the percentage similarity between the conserved regions of the genomes, where the colors vary from yellow (low similarity) to blue (high similarity).

		1	2	3	4	5	6	7	8	9	10	11	12	13	14	15	16	17	18	19	20	21	22	23	24	25	26	27	28	29	30	31	32	33	34	35	36	37	38	39	40	41	42	43	44	45	46	47	48	49	50	51	52	53
1	W. ceti WS74	100	100	100	100	77	77	77	77	77	70	70	70	70	76	76	73	73	73	75	74	74	74	75	75	74	74	77	77	77	77	77	77	77	77	77	77	77	77	77	77	77	77	77	77	77	77	76	77	77	77	77	77	77
2	W. ceti WS105	100	100	100	100	77	77	77	77	77	70	70	70	70	76	76	73	73	73	75	74	74	74	75	75	74	74	77	77	77	77	77	77	77	77	77	77	77	77	77	77	77	77	77	77	77	77	76	77	77	77	77	77	77
3	W. ceti NC36	100	100	100	100	77	77	77	77	77	70	70	70	70	76	76	73	73	73	75	74	74	74	75	75	74	74	77	77	77	77	77	77	77	77	77	77	77	77	77	77	77	77	77	77	77	77	76	77	77	77	77	77	77
4	W. ceti WS08	100	100	100	100	77	77	77	77	77	70	70	70	70	76	76	73	73	73	75	74	74	74	75	75	74	74	77	77	77	77	77	77	77	77	77	77	77	77	77	77	77	77	77	77	77	77	76	77	77	77	77	77	77
5	W. minor DSM20014	77	77	77	77	100	88	87	87	87	71	71	71	71	77	76	74	73	73	75	75	75	75	76	76	75	75	77	77	77	77	77	77	77	77	77	77	77	77	77	77	77	77	77	77	77	77	76	77	77	77	77	77	77
6	W. viridescens NCTC13	77	77	77	77	88	100	99	100	99	72	72	72	72	77	77	75	74	74	76	75	76	76	76	76	76	76	78	78	78	78	78	78	78	78	78	78	78	78	78	78	78	78	78	78	78	78	77	78	78	78	78	78	78
7	W. viridescens NCDO16	77	77	77	77	87	99	100	100	100	71	71	71	71	77	76	74	73	73	76	75	75	75	76	76	75	75	78	78	78	78	78	78	78	78	78	78	78	78	78	78	78	77	78	77	78	78	77	78	78	78	78	78	78
8	W. viridescens DSM204	77	77	77	77	87	100	100	100	100	71	71	71	71	77	77	75	73	73	76	75	75	75	76	76	75	75	78	78	78	78	78	78	78	78	78	78	78	78	78	78	78	78	78	78	78	78	77	78	78	78	78	78	78
9	W. viridescens MFPC16	77	77	77	77	87	99	100	100	100	71	71	71	71	77	77	74	73	73	76	75	75	75	76	76	75	75	78	78	78	78	78	78	78	78	78	78	78	78	78	78	78	78	78	78	78	78	77	78	78	78	78	78	78
10	W. kandleri DSM20593	70	70	70	70	71	71	71	71	71	100	82	82	82	71	71	72	75	75	72	72	72	72	73	73	72	72	74	74	74	74	74	74	74	74	74	74	74	74	74	74	74	74	74	74	74	74	73	74	74	74	74	74	74
11	W. koreensis KACC1551	70	70	70	70	71	72	71	71	71	82	100	100	100	71	71	73	76	76	73	73	73	73	73	73	72	72	75	75	75	75	75	75	75	75	75	75	75	75	75	75	75	75	75	75	75	75	74	75	75	75	75	75	75
12	W. koreensis KCTC3621	70	70	70	70	71	72	71	71	71	82	100	100	100	71	71	73	76	76	73	73	73	73	73	73	72	72	75	75	75	75	75	75	75	75	75	75	75	75	75	75	75	75	75	75	75	75	74	75	75	75	75	75	75
13	W. koreensis WiKim00	70	70	70	70	71	72	71	71	71	82	100	100	100	71	71	73	76	76	73	73	73	73	73	73	72	72	75	75	75	75	75	75	75	75	75	75	75	75	75	75	75	75	75	75	75	75	74	75	75	75	75	75	75
14	W. halotolerans_DSM_	76	76	76	76	77	77	77	77	77	71	71	71	71	100	99	73	73	73	75	75	75	75	75	75	75	75	76	76	76	76	76	76	76	76	76	76	76	76	76	76	76	76	76	76	76	76	75	76	76	76	76	76	76
15	W. halotolerans FBL4	76	76	76	76	76	77	76	77	77	71	71	71	71	99	100	73	73	73	75	74	74	74	75	75	74	75	76	76	76	76	76	76	76	76	76	76	76	76	76	76	76	76	76	76	76	76	75	76	76	76	76	76	76
16	W. oryzae SG25	73	73	73	73	74	75	74	75	74	72	73	73	73	73	73	100	76	76	77	77	77	77	77	77	77	77	81	81	81	81	81	81	81	81	81	81	81	81	81	81	81	81	81	81	81	81	80	81	81	81	81	81	81
17	W. soli CECT7031	73	73	73	73	73	74	73	73	73	75	75	75	75	73	73	75	100	100	76	76	76	76	76	76	76	76	79	79	79	79	79	79	79	79	79	79	79	79	79	79	79	79	79	79	79	79	78	79	79	79	79	79	79
18	W. soli KACC11848	73	73	73	73	73	74	73	73	73	75	75	75	75	73	73	75	100	100	76	76	76	76	76	76	76	76	79	79	79	79	79	79	79	79	79	79	79	79	79	79	79	79	79	79	79	79	78	79	79	79	79	79	79
19	W. bombi R53094	75	75	75	75	75	76	76	76	76	72	73	73	73	75	75	77	76	76	100	95	95	95	91	91	91	91	84	84	84	84	84	84	84	84	84	84	84	84	84	84	84	84	84	84	84	84	83	84	84	84	84	84	84
20	W. hellenica R53116	74	74	74	74	75	76	75	75	75	72	73	73	73	75	74	77	76	76	95	100	98	98	91	91	90	90	84	84	84	84	84	84	84	84	84	84	84	84	84	84	84	84	84	84	84	84	82	83	84	84	84	84	84
21	W. hellenica 0916-4-2	74	74	74	74	75	76	75	75	75	72	73	73	73	75	74	77	76	76	95	98	100	100	91	91	90	90	84	84	84	84	84	84	84	84	84	84	84	84	84	84	84	84	84	84	84	84	83	84	84	84	84	84	84
22	W. hellenica Wikim14	74	74	74	74	75	76	75	75	75	72	73	73	73	75	74	77	76	76	95	98	100	100	91	91	90	90	84	84	84	84	84	84	84	84	84	84	84	84	84	84	84	84	84	84	84	84	83	84	84	84	84	84	84
23	W. paramesenteroides	75	75	75	75	76	76	76	76	76	73	73	73	73	75	75	77	76	76	91	91	91	91	100	100	95	95	85	85	85	85	85	85	85	85	85	85	85	85	85	85	85	85	85	85	85	85	84	85	85	85	85	85	85
24	W. paramesenteroides	75	75	75	75	76	76	76	76	76	73	73	73	73	75	75	77	76	76	91	91	91	91	100	100	95	95	85	85	85	85	85	85	85	85	85	85	85	85	85	85	85	85	85	85	85	85	84	85	85	85	85	85	85
25	W. jogaejeotgali FOL01	74	74	74	74	75	76	75	75	75	72	72	72	72	75	74	77	76	76	91	90	90	90	95	95	100	99	84	84	84	84	84	84	84	84	84	84	84	84	84	84	84	84	84	84	84	84	83	84	84	84	84	84	84
26	W. thailandensis KCTC	74	74	74	74	75	76	75	75	75	72	72	72	72	75	75	77	76	76	91	90	90	90	95	95	99	100	84	84	84	84	84	84	84	84	84	84	84	84	84	84	84	84	84	84	84	84	83	84	84	84	84	84	84
27	W. cibaria BM2	77	77	77	77	77	78	78	78	78	74	75	75	75	76	76	81	79	79	84	84	84	84	85	85	84	84	100	100	100	100	100	100	100	100	100	100	100	100	100	100	100	100	100	100	99	100	93	94	95	95	95	95	95
28	W. cibaria KACC11862	77	77	77	77	77	78	78	78	78	74	75	75	75	76	76	81	79	79	84	84	84	84	85	85	84	84	100	100	100	100	100	100	100	100	100	100	100	100	100	100	100	100	100	100	100	100	93	95	95	95	95	95	95
29	W. cibaria CMU	77	77	77	77	77	78	78	78	78	74	75	75	75	76	76	81	79	79	84	84	84	84	85	85	84	84	100	100	100	100	100	100	100	100	100	100	100	100	100	100	100	100	100	100	100	100	93	95	95	95	95	95	95
30	W. cibaria CMS3	77	77	77	77	77	78	78	78	78	74	75	75	75	76	76	81	79	79	84	84	84	84	85	85	84	84	100	100	100	100	100	100	100	100	100	100	100	100	100	100	100	100	100	100	100	100	93	95	95	95	95	95	95
31	W. cibaria CMS1	77	77	77	77	77	78	78	78	78	74	75	75	75	76	76	81	79	79	84	84	84	84	85	85	84	84	100	100	100	100	100	100	100	100	100	100	100	100	100	100	100	100	100	100	100	100	93	95	95	95	95	95	95
32	W. cibaria CMS2	77	77	77	77	77	78	78	78	78	74	75	75	75	76	76	81	79	79	84	84	84	84	85	85	84	84	100	100	100	100	100	100	100	100	100	100	100	100	100	100	100	100	100	100	100	100	93	95	95	95	95	95	95
33	W. cibaria OF06-4	77	77	77	77	77	78	78	78	78	74	75	75	75	76	76	81	79	79	84	84	84	84	85	85	84	84	100	100	100	100	100	100	100	100	100	100	100	100	100	100	100	100	100	100	99	100	93	95	95	95	95	95	95
34	W. cibaria DmW103	77	77	77	77	77	78	78	78	78	74	75	75	75	76	76	81	79	79	84	84	84	84	85	85	84	84	100	100	100	100	100	100	100	100	100	100	100	100	100	100	100	100	100	100	99	100	93	95	95	95	95	95	95
35	W. cibaria AM27-24	77	77	77	77	77	78	78	78	78	74	75	75	75	76	76	81	79	79	84	84	84	84	85	85	84	84	100	100	100	100	100	100	100	100	100	100	100	100	100	100	100	100	100	100	100	100	93	95	95	95	95	95	95
36	W. cibaria AM27-22LB	77	77	77	77	77	78	78	78	78	74	75	75	75	76	76	81	79	79	84	84	84	84	85	85	84	84	100	100	100	100	100	100	100	100	100	100	100	100	100	100	100	100	100	100	100	100	93	95	95	95	95	95	95
37	W. cibaria M2	77	77	77	77	77	78	78	78	78	74	75	75	75	76	76	81	79	79	84	84	84	84	85	85	84	84	100	100	100	100	100	100	100	100	100	100	100	100	100	100	100	100	100	100	99	100	93	95	95	95	95	95	95
38	W. cibaria CH2	77	77	77	77	77	78	78	78	78	74	75	75	75	76	76	81	79	79	84	84	84	84	85	85	84	84	100	100	100	100	100	100	100	100	100	100	100	100	100	100	100	100	100	100	99	100	93	95	95	95	95	95	95
39	W. cibaria UBA11294	77	77	77	77	77	78	78	78	78	74	75	75	75	76	76	81	79	79	84	84	84	84	85	85	84	84	100	100	100	100	100	100	100	100	100	100	100	100	100	100	100	100	100	100	99	100	93	95	95	95	95	95	95
40	W. cibaria ff3PR	77	77	77	77	77	78	78	78	78	74	75	75	75	76	76	81	79	79	84	84	84	84	85	85	84	84	100	100	100	100	100	100	100	100	100	100	100	100	100	100	100	100	100	100	99	99	93	95	95	95	95	95	95
41	W. cibaria MG1	77	77	77	77	77	78	78	78	78	74	75	75	75	76	76	81	79	79	84	84	84	84	85	85	84	84	100	100	100	100	100	100	100	100	100	100	100	100	100	100	100	100	100	100	99	99	93	95	95	95	95	95	95
42	W. cibaria UBA11274	77	77	77	77	77	78	77	78	78	74	75	75	75	76	76	81	79	79	84	84	84	84	85	85	84	84	100	100	100	100	100	100	100	100	100	100	100	100	100	100	100	100	100	100	99	99	93	94	95	95	95	95	95
43	W. cibaria FBL5	77	77	77	77	77	78	78	78	78	74	75	75	75	76	76	81	79	79	84	84	84	84	85	85	84	84	100	100	100	100	100	100	100	100	100	100	100	100	100	100	100	100	100	100	99	99	93	94	95	95	95	95	95
44	W. cibaria AB3b	77	77	77	77	77	78	77	78	78	74	75	75	75	76	76	81	79	79	84	84	84	84	85	85	84	84	100	100	100	100	100	100	100	100	100	100	100	100	100	100	100	100	100	100	99	99	93	94	95	95	95	95	95
45	Weissella sp DD23	77	77	77	77	77	78	78	78	78	74	75	75	75	76	76	81	79	79	84	84	84	84	85	85	84	84	99	100	100	100	100	100	99	99	100	100	99	99	99	99	99	99	99	99	100	99	93	94	95	95	95	95	95
46	W. cibaria 110	77	77	77	77	77	78	78	78	78	74	75	75	75	76	76	81	79	79	84	84	84	84	85	85	84	84	100	100	100	100	100	100	100	100	100	100	100	100	100	99	99	99	99	99	99	100	93	95	95	95	95	95	95
47	W. confusa UBA11342	76	76	76	76	76	77	77	77	77	73	74	74	74	75	75	80	79	79	83	82	83	83	84	84	83	83	93	93	93	93	93	93	93	93	93	93	93	93	93	93	93	93	93	93	93	93	100	98	98	98	98	98	98
48	W. confusa MBF8-1	77	77	77	77	77	78	78	78	78	74	75	75	75	76	76	81	80	80	84	83	84	84	85	85	84	84	94	95	95	95	95	95	95	95	95	95	95	95	95	95	95	94	94	94	94	95	98	100	100	100	100	100	100
49	W. confusa AF03-13	77	77	77	77	77	78	78	78	78	74	75	75	75	76	76	81	80	80	84	84	84	84	85	85	84	84	95	95	95	95	95	95	95	95	95	95	95	95	95	95	95	95	95	95	95	95	98	100	100	100	100	100	100
50	W. confusa LBAE-C39-	77	77	77	77	77	78	78	78	78	74	75	75	75	76	76	81	80	80	84	84	84	84	85	85	84	84	95	95	95	95	95	95	95	95	95	95	95	95	95	95	95	95	95	95	95	95	98	100	100	100	100	100	100
51	W. confusa 32	77	77	77	77	77	78	78	78	78	74	75	75	75	76	76	81	80	80	84	84	84	84	85	85	84	84	95	95	95	95	95	95	95	95	95	95	95	95	95	95	95	95	95	95	95	95	98	100	100	100	100	100	100
52	W. confusa AB3E41	77	77	77	77	77	78	78	78	78	74	75	75	75	76	76	81	80	80	84	84	84	84	85	85	84	84	95	95	95	95	95	95	95	95	95	95	95	95	95	95	95	95	95	95	95	95	98	100	100	100	100	100	100
53	W. confusa DSM20196	77	77	77	77	77	78	78	78	78	74	75	75	75	76	76	81	80	80	84	84	84	84	85	85	84	84	95	95	95	95	95	95	95	95	95	95	95	95	95	95	95	95	95	95	95	95	98	100	100	100	100	100	100
